# Efficacy and Safety of Farletuzumab in Ovarian Cancer: A Systematic Review and Single-Arm Meta-Analysis

**DOI:** 10.7759/cureus.73503

**Published:** 2024-11-12

**Authors:** Aya M Fayoud, Moaz Yasser Darwish, Eman Ayman Nada, Abdallah A Helal, Nada Shaaban Mohamed, Asmaa Ahmed Elrashedy, Mohamed Abd-ElGawad

**Affiliations:** 1 Faculty of Pharmacy, Kafr El-Shaikh University, Kafr El-Shaikh, EGY; 2 Faculty of Medicine, Fayoum University, Fayoum, EGY; 3 Faculty of Pharmacy, Tanta University, Tanta, EGY; 4 Faculty of Medicine, Kafr El-Shaikh University, Kafr El-Shaikh, EGY; 5 Department of Neurology, Faculty of Medicine, Fayoum University, Fayoum, EGY

**Keywords:** farletuzumab, frα, humanized monoclonal antibody, oncology, ovarian cancer

## Abstract

Folate receptor alpha (FRα) has emerged as a promising target in the treatment of ovarian cancer, with farletuzumab, a humanized monoclonal antibody targeting FRα, showing potential in clinical settings. This systematic review and single-arm meta-analysis aimed to evaluate the efficacy and safety of farletuzumab in patients with solid tumors, particularly ovarian cancer. Following the Preferred Reporting Items for Systematic Reviews and Meta-Analyses (PRISMA) guidelines, we conducted a thorough search across PubMed, the Web of Science, Scopus, and the Cochrane Central Register of Controlled Trials (CENTRAL) for clinical trials assessing farletuzumab in solid tumors. Data were extracted on study characteristics, patient demographics, treatment regimens, and efficacy outcomes including progression-free survival (PFS), overall survival (OS), response rates, and adverse events (AEs). The pooled analyses were performed using the Open Meta-Analyst software.

In total, seven prospective studies were included, covering various farletuzumab regimens in ovarian cancer and other solid tumors. The pooled PFS was 10.5 months (95% CI: 8, 15.7) across three studies involving 925 patients, while the pooled OS was 36.7 months (95% CI: 26.6, 35) in two studies with 881 patients. Treatment response rates indicated a partial response in 55.25% of patients and stable disease in 28.68% of cases. Gastrointestinal and hematological AEs were frequently reported, with nausea (52.14%), neutropenia (50.65%), and anemia (39.76%) being the most common.

Farletuzumab appears to offer a promising efficacy profile, particularly in ovarian cancer, with notable improvements in disease progression and survival. However, the treatment is associated with a high incidence of gastrointestinal and hematological AEs, raising the need for careful patient selection. Further studies are required to refine the therapeutic regimen and ensure an optimal balance between efficacy and safety.

## Introduction and background

Folate receptor alpha (FRα), a glycosylphosphatidylinositol (GPI)-anchored cell surface glycoprotein encoded by the FOLR1 gene, exhibits a high affinity for folic acid and its derivatives [1], crucial for cellular processes like cell division and proliferation and tissue growth through signaling cascades and components of the folate cycle [2-4].

While FRα expression in normal tissues is limited, it becomes more widespread in various cancers, including ovarian, endometrial, lung, and breast cancer subsets [5-7]. Notably, FRα is overexpressed in up to 90% of ovarian cancers, though expression levels vary across different histotypes [8-10]. Tumor cells can release a soluble form of FRα into circulation, known as sFRα [11-13], making it a potential clinical biomarker for ovarian cancer.

Ovarian cancer, ranking as the eighth most common cancer in women and 18th overall, accounted for over 313,959 new cases in 2020 [14]. The challenge of often diagnosing ovarian cancer at advanced stages, coupled with acquired drug resistance and limited effective initial treatments, underscores the need for innovative interventions [15].

Farletuzumab, a humanized monoclonal antibody targeting FRα, has shown promise in preclinical models of ovarian cancer. Studies demonstrate its antitumor effects, both as a monotherapy and in combination with other chemotherapy agents [16]. Encouraging results, particularly in patients with low baseline cancer antigen 125 (CA-125), were observed in platinum-sensitive ovarian cancer when farletuzumab was administered alongside typical chemotherapy drugs [17,18].

Despite diverse studies approaching intervention from various perspectives, a lack of high-level evidence persists. This systematic review and single-arm meta-analysis aims to explore and provide insights into the efficacy and safety of farletuzumab in patients with solid tumors, primarily ovarian cancer.

## Review

Methodology

We adhered to the Preferred Reporting Items for Systematic Reviews and Meta-Analyses (PRISMA) statement guidelines [19] and followed recommendations from the Cochrane Handbook for Systematic Reviews and Meta-Analyses [20].

Data Sources and Search Strategy

We conducted a comprehensive search across PubMed, the Web of Science, Scopus, and the Cochrane Central Register of Controlled Trials (CENTRAL) databases up to April 1, 2024. Our search strategy is as follows: (Farletuzumab OR MORAb-003 OR MORAb-202) AND (Tumor OR Neopla OR Cancer OR malign* OR carcinoma OR sarcoma OR Metas*). The search results are the following: PubMed: 54 records, Web of Science: 112 records, Scopus: 194 records, and CENTRAL: 24 records.

The search combined general terms for malignancies and specific terms relevant to farletuzumab to ensure comprehensive inclusion of studies, recognizing that many trials might involve broader cancer populations while assessing outcomes for ovarian cancer.

Eligibility Criteria

We included clinical trials (randomized or non-randomized) that assessed farletuzumab for any malignant solid tumors, with no specific control group required. We excluded observational studies, study protocols, editorials, non-English publications, and non-human studies. The population, intervention, comparison, and outcome (PICO) criteria are as follows: (P) patients with malignant solid tumors, (I) farletuzumab, (C) any, and (O) progression-free survival (PFS) and overall survival (OS) as the primary outcomes in addition to response rates such as stable disease, progressive disease, complete response, partial response, and the incidence of adverse events (AEs).

Data Extraction and Management

The data extraction was performed by two authors independently, resolving discrepancies through discussion. The extracted data included basic trial information (e.g., author, year, sample size), patient demographics, treatment details, and clinical outcomes such as PFS, OS, and the occurrence of AEs. Extraction forms were standardized and reviewed by the first author for accuracy.

Quality Assessment

We used the ROBINS-I tool to assess the risk of bias for non-randomized studies [21]. This tool evaluates seven bias domains, providing a robust framework for identifying potential study biases. For randomized trials, the Cochrane risk-of-bias tool was used where applicable.

Statistical Analysis

Meta-analysis was performed using the Open Meta-Analyst (OMA) software. We pooled the incidence of AEs and calculated survival rates with 95% confidence intervals. Sensitivity analysis was conducted to explore sources of heterogeneity, particularly when I² exceeded 50%.

Result

Literature Search Results

**Figure 1:**
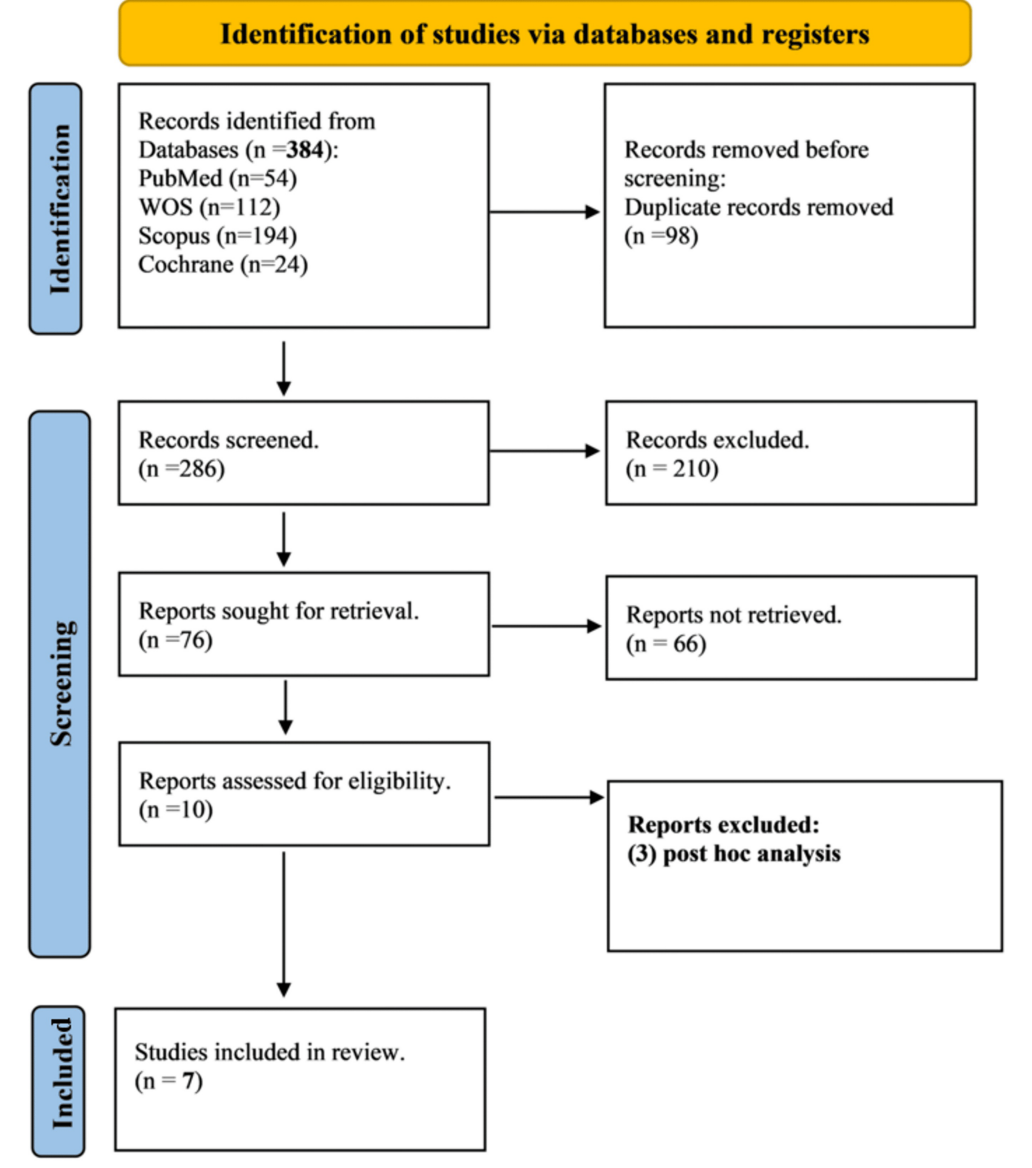
PRISMA flow diagram of the systematic review. PRISMA: Preferred Reporting Items for Systematic Reviews and Meta-Analyses

Our systematic search identified 384 potential studies. After eliminating 98 duplicates, a further exclusion of 286 studies occurred during title/abstract screening. Subsequent full-text screening resulted in the exclusion of an additional 69 studies. Ultimately, seven studies met the eligibility criteria for inclusion in both the quantitative and qualitative synthesis of this systematic review (Figure 1; PRISMA).

Quality Assessment

Five studies including Armstrong et al., Kim et al., Konner et al., Sasaki et al., and Shimizu et al. [22-25,17] were identified as having a potential for bias due to confounding. This was primarily attributed to the nature of the studies being open-lab and single-arm, lacking a division of participants' follow-up time based on the interventions they received. Additionally, there was an absence of information on whether any variables post-intervention, which could influence the results, were controlled by the authors.

Regarding bias due to missing data, specifically, Armstrong et al. provided no details on whether participants were excluded because of missing data related to their intervention status or other critical variables necessary for the analysis.

**Figure 2:**
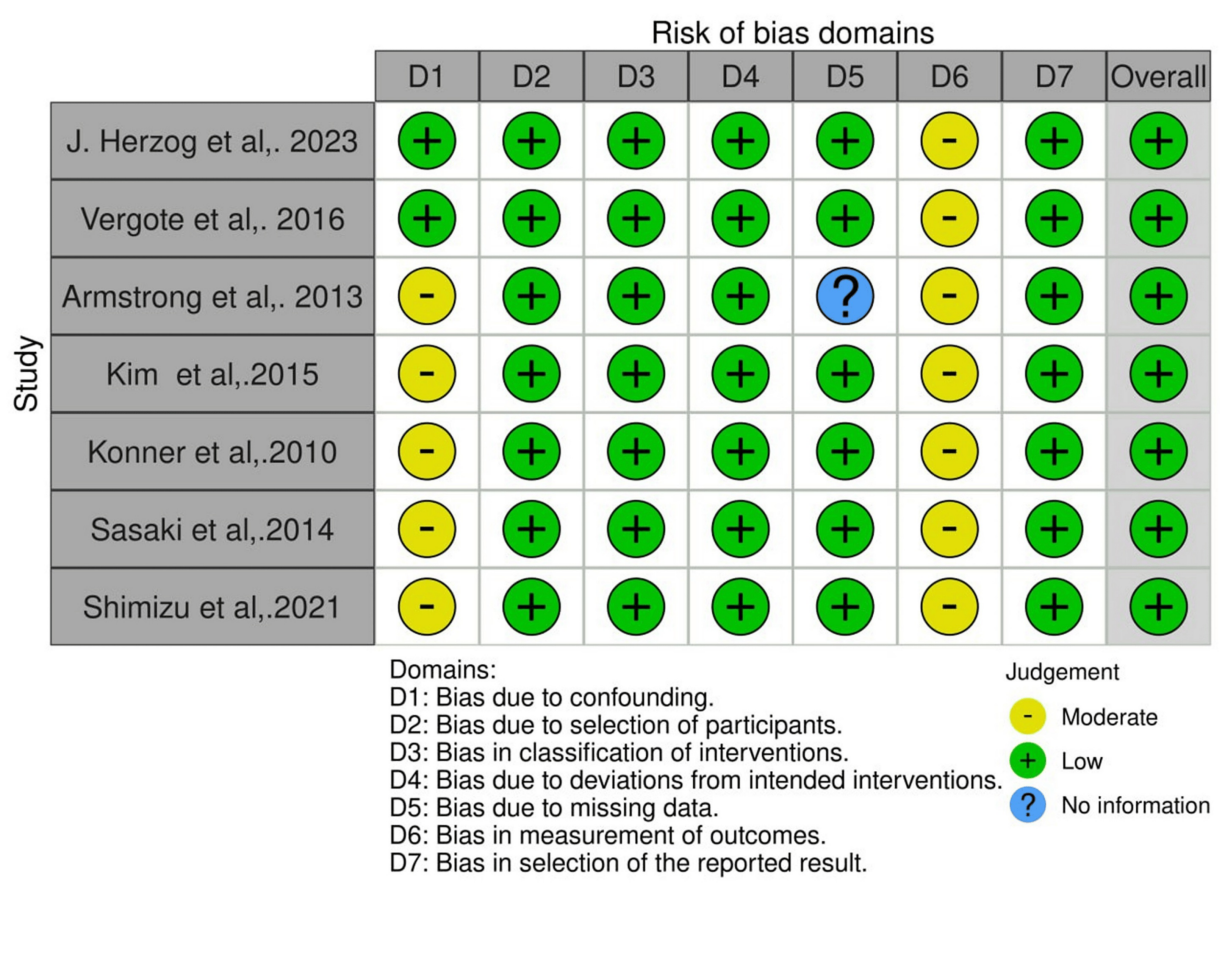
ROBINS-I quality assessment graph of the included studies.

For the bias in the measurement of outcomes, the same set of studies [22-25,17] was evaluated to have a moderate risk of bias. This assessment stemmed from the possibility that the measurement of outcomes could have been inadvertently influenced by the participants' and outcome assessors' awareness of the intervention status. Furthermore, the methodologies applied in assessing outcomes were not uniformly applied across different intervention groups. Despite these concerns, the overall bias across all studies was considered low (Figure 2).

Characteristics of the Included Studies

This meta-analysis included seven prospective studies, all contributing valuable insights. These study a range of farletuzumab interventions, with two focusing on farletuzumab monotherapy, three exploring combinations with carboplatin and taxane (paclitaxel, docetaxel), one investigating its conjunction with carboplatin and pegylated liposomal doxorubicin (PLD), and the final one associating farletuzumab with eribulin mesylate. Notably, four studies specifically targeted platinum-sensitive patients.

The study population predominantly comprised women diagnosed with ovarian cancer, with minimal representation of men afflicted by other solid tumors such as gastric and lung carcinoma. Farletuzumab was administered intravenously in all studies, with varying doses tailored to individual body weight or surface area. The standard administration protocol involved a six-cycle regimen at three-week intervals (refer to Table 1 for specific details).

**Table 1:** Summary of the clinical trials eligible for enrollment. IV: intravenously; AUC: area under the curve

Study ID	Country	Design/phases	Total sample size in the study	Disease	Intervention								
					Name and doses				Combined medications	Number of participants in the group			Regimen
Shimizu et al., 2021 [25]	Japan	Open-label, first-in-human, single-arm dose escalation phase 1 trial	22	Folate receptor-a-positive advanced solid tumors (breast (n=2), endometrial (n=3), non-small cell lung carcinoma (n=4), ovarian (n=12), and fallopian tube (n=1))	IV farletuzumab (MORAb-202): 0.3-1.2 mg/kg once every three weeks				Farletuzumab was linked to eribulin mesylate	22			Dose escalation (patients received MORAb-202 IV at doses of 0.3-1.2 mg/kg once every three weeks)
Sasaki et al., 2015 [24]	Japan	Open-label, single-arm dose escalation phase I trial	16	Solid tumors (ovarian cancer (n=14) and gastric cancer (n=2))	IV farletuzumab (MORAb-003): 50, 100, 200, and 400 mg/m^2^					16			The starting dose of farletuzumab was decided to be 50 mg/m^2^. Dose escalation was followed by 100, 200, and 400 mg/ m^2^ doses on days 1, 8, 15, and 22 once a week for four weeks as one cycle
Konner et al., 2010 [23]	United States	Open-label, single-arm dose escalation study/phase 1	25	Epithelial ovarian cancer	IV farletuzumab (MORAb-003): 12.5-400 mg/m^2^					25			Dose escalation followed a modified Fibonacci sequence incorporating the following dose levels: 12.5, 25, 37.5, 62.5, 100, 200, and 400 mg/m^2^ on days 1, 8, 15, and 22 of a five-week cycle
Kim et al., 2016 [22]	United States	Multicenter, single-arm, open-label phase 1b study	15	Platinum-sensitive epithelial ovarian cancer	IV farletuzumab (2.5 mg/kg)				Carboplatin and pegylated liposomal doxorubicin	15			Weekly farletuzumab 2.5 mg/kg plus carboplatin AUC 5-6 and pegylated liposomal doxorubicin 30 mg/m^2^ every four weeks for six cycles. Subsequently, maintenance with single-agent farletuzumab 2.5 mg/kg once weekly or farletuzumab 7.5 mg/kg once every three weeks continued until progression
Armstrong et al., 2013 [17]	16 centers in North America and Europe	Multicenter, open-label clinical trial	54	First relapsed ovarian cancer platinum-sensitive low CA-125, (non-mucinous ovarian, fallopian tube, or primary peritoneal cancer)	IV farletuzumab (MORAb-003)	37.5 mg/m^2^ weekly	62.5 mg/m^2^ weekly	100 mg/m^2^ weekly	Carboplatin plus taxane (paclitaxel, docetaxel)	(4) 37.5 mg/m^2^ weekly	(5) 62.5 mg/m^2^ weekly	(45) 100 mg/m^2^ weekly	A single agent or combined with carboplatin (AUC 5-6) and taxane (paclitaxel 175 mg/m^2^ or docetaxel 75 mg/m^2^), every 21 days for six cycles, followed by farletuzumab maintenance until progression
Vergote et al., 2016 [18]	North America, Europe, the Asia-Pacific region, Latin America, Japan	Multicenter, double-blind, randomized placebo-controlled phase III	1102	First relapsed ovarian cancer platinum-sensitive low CA-125 (non-mucinous epithelial ovarian cancer (1091))	Intraperitoneal or IV farletuzumab (MORAb-003)	1.25 mg/kg weekly	2.5 mg/kg weekly		Carboplatin plus taxane (paclitaxel, docetaxel)	(370) 1.25 mg/kg weekly	(366) 2.5 mg/kg weekly		Six cycles with carboplatin (target AUC, 5-6) administered IV and the investigator's choice of taxane (paclitaxel 175 mg/m^2^ or docetaxel 75 mg/m^2^) IV every three weeks. Patients were randomly assigned to receive weekly farletuzumab 1.25 mg/kg or farletuzumab 2.5 mg/kg
Herzog et al., 2023 [26]	United States, Europe, Japan	Multicenter, double-blind, randomized placebo-controlled phase II	214	First relapsed ovarian cancer platinum-sensitive low CA-125 (solid tumors: ovarian tumors (117), fallopian tube (15), primary peritoneal (9), and missing (1))	IV farletuzumab (MORAb-003)	10 mg/kg loading dose with a 5 mg/kg weekly			Carboplatin plus paclitaxel or carboplatin plus pegylated liposomal doxorubicin				Six cycles of open-label treatment with either carboplatin (AUC 5) plus paclitaxel (175 mg/m^2^ IV every three weeks) or carboplatin (AUC 5) plus pegylated liposomal doxorubicin (30 mg/m^2^ IV every four weeks) in combination with a blinded assignment to either farletuzumab or placebo. All patients received a loading dose of 10 mg/kg farletuzumab (or placebo) for the first two weeks, followed by 5 mg/kg weekly

The mean age of patients ranged from 55.2 to 63.2 years. Four studies reported Eastern Cooperative Oncology Group (ECOG) performance status, indicating that 180 patients scored 0, while 53 patients scored 1. Additionally, three studies provided insights into the duration of the first remission, ranging from six to 12 months for 411 patients and exceeding 18 months for 149 patients. The studies comprehensively reported data on OS, PFS, and the occurrence of AEs, with detailed information available in Table 2.

**Table 2:** Information on the clinical trials eligible for enrollment. CR: complete response; PR: partial response; SD: stable disease; PFS: progression-free survival; OS: overall survival; 95% CI: 95% confidence intervals; NA: not available; ECOG: Eastern Cooperative Oncology Group

Study ID	Total	Age (year) mean (SD)	Sex male/female	ECOG performance status		First remission length (months)			PFS median (95% CI) (months)	OS median (95% CI) (months)	SD	PD	CR	PR
				0	1	6-12 months	12-18 months	>18 months						
Herzog et al., 2023 [26]	142	63 (9.33)	0/142	118	23	NA	NA	NA	11.7 (10.2, 13.6)	43.07 (37.1, -)	36	5	24	72
Vergote et al., 2016 [18]	736	58 (9.05)	0/736	NA	NA	389	223	124	9.6 (8.6, 10.4)	30.4 (26.6, 35)	NA	NA	NA	NA
Armstrong et al., 2013 [17]	54	63.2 (11.7)	0/54	36	18	16	14	17	10.3 (8.0, 15.7)	NA	9	2	3	30
Kim et al., 2016 [22]	15	63.25 (10.12)	0/15	NA	NA	6	1	8	NA	NA	4	NA	1	10
Konner et al., 2010 [23]	25	58.75 (8.75)	0/25	NA	NA	NA	NA	NA	NA	NA	9	15	NA	NA
Sasaki et al., 2015 [24]	16	55.25 (9.25)	2/14	11	5	NA	NA	NA	NA	NA	8	7	NA	NA
Shimizu et al., 2021 [25]	22	56 (14.26)	2/20	15	7	NA	NA	NA	NA	NA	8	4	1	9

Efficacy of Farletuzumab

The pooled PFS was 10.5 months (95% CI: 8, 15.7) across three studies involving 925 patients. Furthermore, the OS was 36.7 months (95% CI: 26.6, 35) based on two studies with 881 patients.

**Figure 3:**
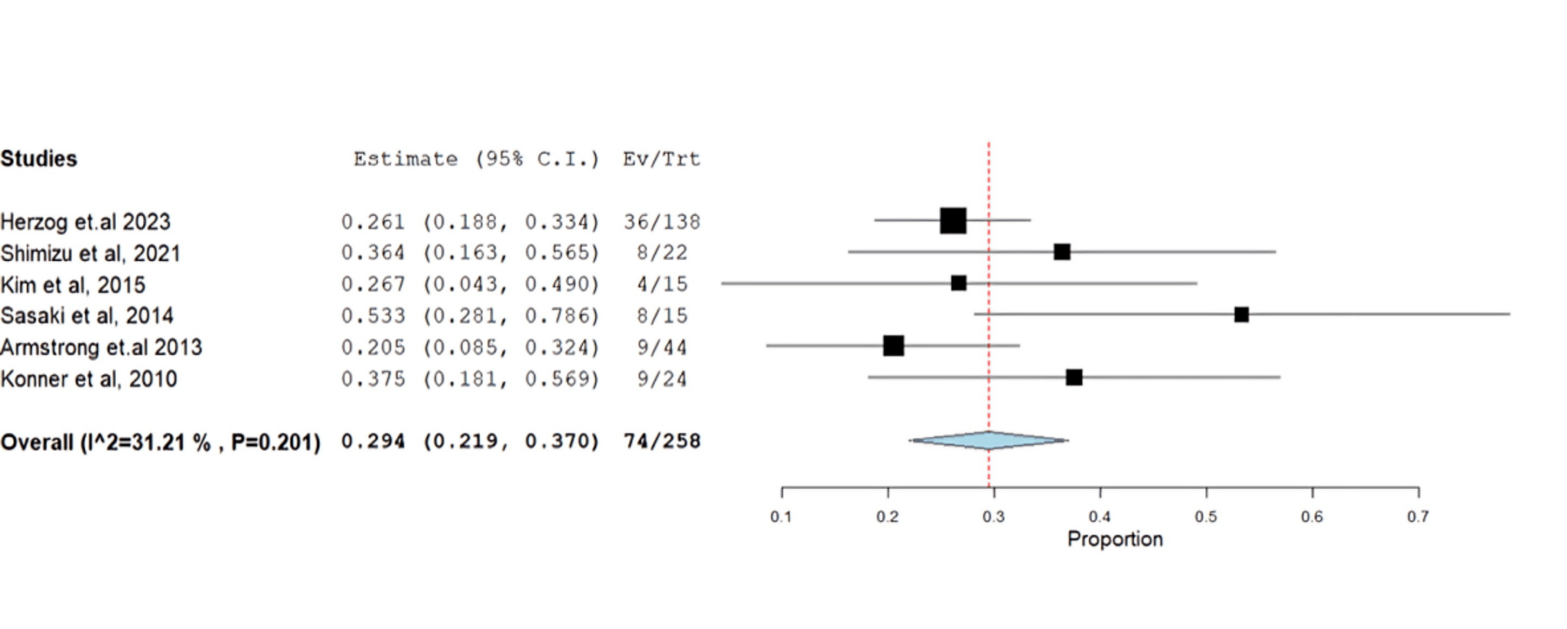
Forest plot of stable disease.

**Figure 4:**
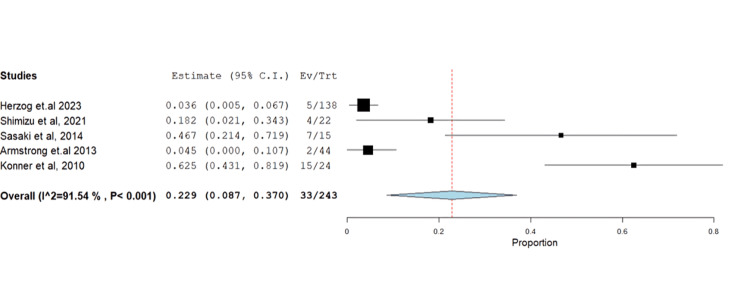
Forest plot of progressive disease.

**Figure 5:**
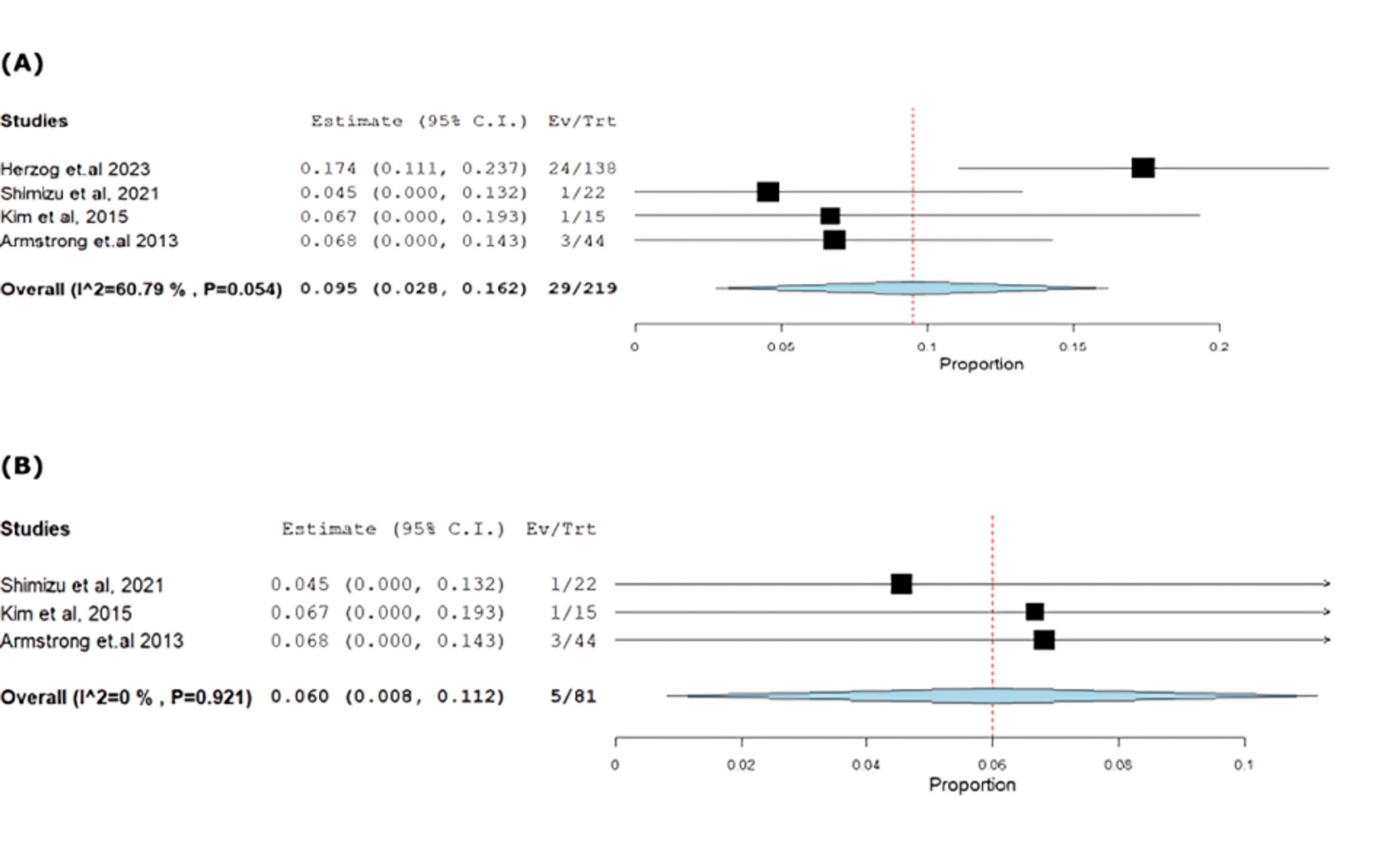
(A) Forest plot of complete response. (B) Sensitivity analysis of complete response.

**Figure 6:**
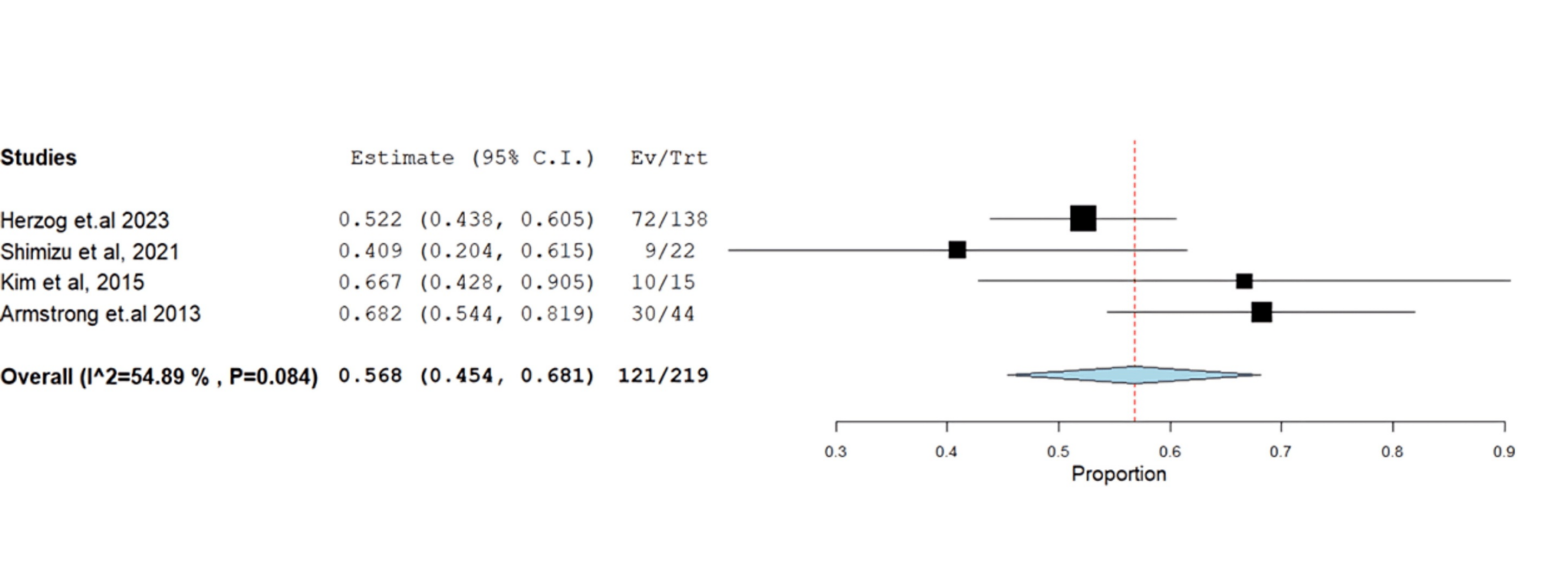
Forest plot of partial response disease.

The impact of farletuzumab on different disease outcomes was examined, revealing incidence for stable disease, progressive disease, complete response, and partial response of 28.68% (95% CI: 0.21-0.37), 13.58% (95% CI: 0.08, 0.37), 13.24% (95% CI: 0.02, 0.16), and 55.25% (95% CI: 0.45, 0.68), respectively. Detailed data can be found in Table 3 and Table 4, with corresponding figures provided in Figures 3-6.

**Table 3:** Efficacy of farletuzumab. CR: complete response; PR: partial response; SD: stable disease; PFS: progression-free survival; OS: overall survival; 95% CI: 95% confidence intervals; NA: not available

Study ID	PFS			OS			SD		PD		CR		PRD	
	Median (months)	95% CI	Total	Median (months)	95% CI	Total	Event	Total	Event	Total	Event	Total	Event	Total
Herzog et al., 2023 [26]	11.7	10.2, 13.6	142	43.07	37.1, −	142	36	138	5	138	24	138	72	138
Shimizu et al., 2021 [25]	NA	NA	NA	NA	NA	NA	8	22	4	22	1	22	9	22
Vergote et al., 2016 [18]	9.6	8.6, 10.4	739	30.4	26.6, 35	739	NA	NA	NA	NA	NA	NA	NA	NA
Kim et al., 2016 [22]	NA	NA	NA	NA	NA	NA	4	15	NA	NA	1	15	10	15
Sasaki et al., 2015 [24]	NA	NA	NA	NA	NA	NA	8	15	7	15	NA	NA	NA	NA
Armstrong et al., 2013 [17]	10.3	8.0, 15.7	44	NA	NA	NA	9	44	2	44	3	44	30	44
Konner et al., 2010 [23]	NA	NA	NA	NA	NA	NA	9	24	15	24	NA	NA	NA	NA

**Table 4:** Efficacy of farletuzumab (continuation). SE: standard error

Variables	No. of studies	Effect estimates	Incidence %	95% CI	SE	P-value	No. of participants	Heterogeneity	
								I^2^	P-value
Stable disease	6	0.294	28.68	(0.219-0.370)	0.038	<0.001	74	31.212	0.201
Progressive disease	5	0.229	13.58	(0.087-0.370)	0.072	0.002	33	91.541	<0.001
Complete response	4	0.095	13.24	(0.028-0.162)	0.034	0.006	29	60.794	0.054
Partial response disease	4	0.568	55.25	(0.454-0.681)	0.058	<0.001	121	54.889	0.084

AEs of Farletuzumab

**Figure 7:**
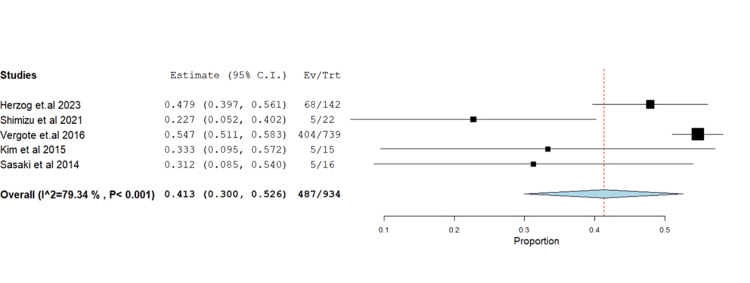
Forest plot of nausea.

Concerning toxicity, gastrointestinal AEs were prominent, including nausea (52.14%, 95% CI: 0.30-0.52). The pooled results exhibited heterogeneity (p<0.001, I^2^=79.3%) without resolution (Figure 7).

**Figure 8:**
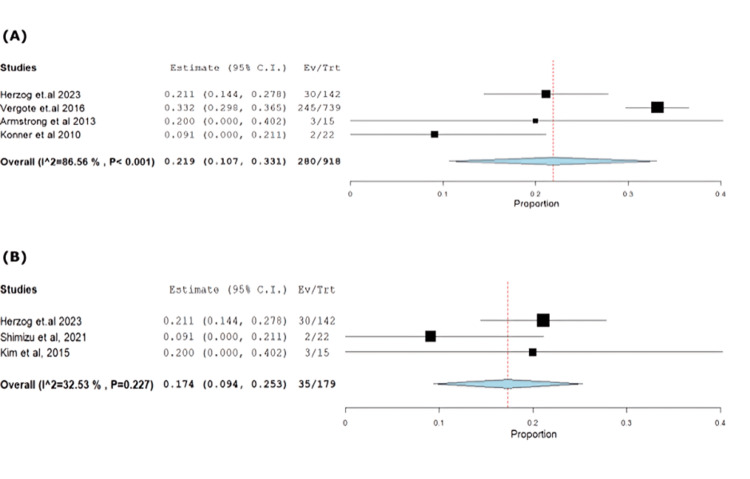
(A) Forest plot of vomiting. (B) Sensitivity analysis of vomiting.

Vomiting occurred at 30.5% (95% CI: 0.10-0.33) with heterogeneous results (p<0.001, I^2^=86.5%). Exclusion of Vergote et al. [18] resolved heterogeneity (p=0.22, I^2^=32.5%) (Figure 8A-8B).

**Figure 9:**
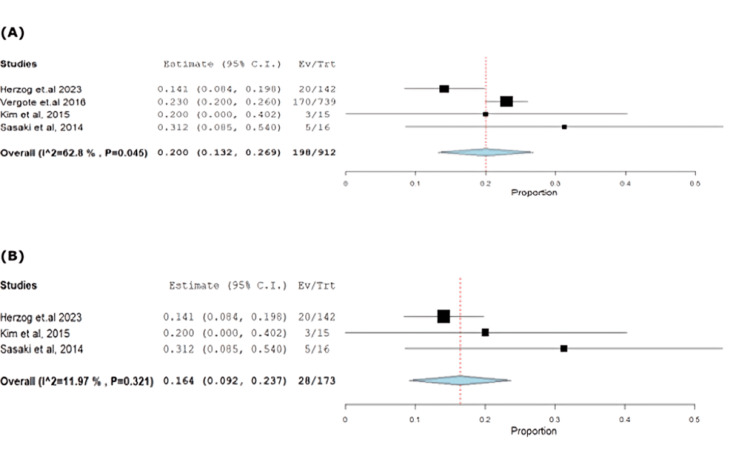
(A) Forest plot of decreased appetite. (B) Sensitivity analysis of decreased appetite.

Similarly, decreased appetite was observed at 21.71% (95% CI: 0.13-0.26), with heterogeneous results (p=0.045, I^2^=62.7%). Exclusion of Vergote et al. [18] resolved heterogeneity (p=0.32, I^2^=11.9%) (Figure 9A-9B).

**Figure 10:**
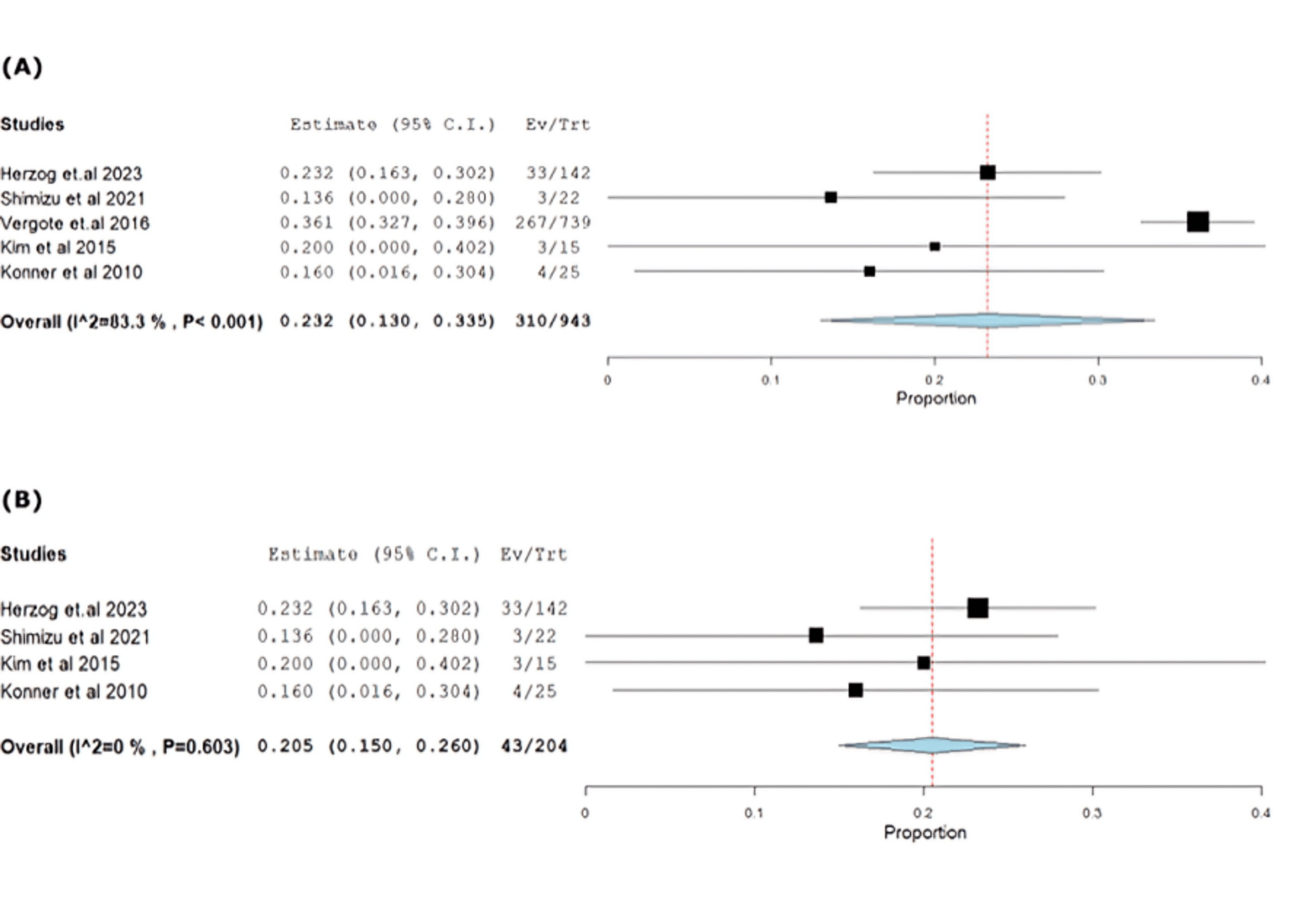
(A) Forest plot of diarrhea. (B) Sensitivity analysis of diarrhea.

Diarrhea incidence was 32.87% (95% CI: 0.13-0.33), showing heterogeneity (p<0.001, I^2^=83.2%), resolved by excluding Vergote et al. [18] (p=0.6, I^2^=0%) (Figure 10A-10B).

**Figure 11:**
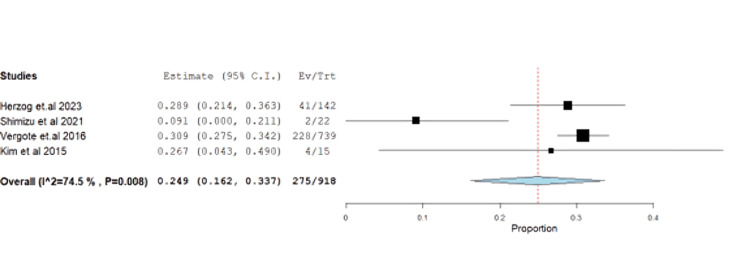
Forest plot of constipation.

Constipation was reported at 29.95% (95% CI: 0.16-0.33) with unresolved heterogeneity (p=0.008, I^2^=74.5%) (Figure 11).

**Figure 12:**
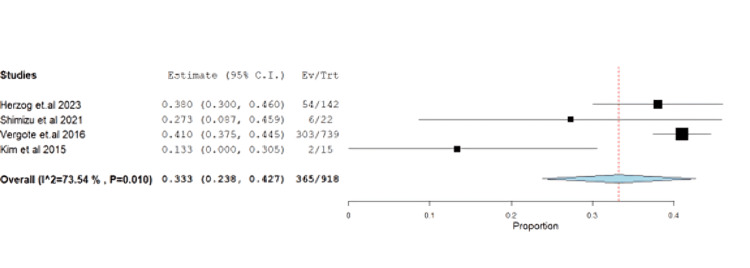
Forest plot of anemia.

Hematological AEs showed anemia at 39.76% (95% CI: 0.23-0.42) with unresolved heterogeneity (p=0.01, I^2^=73.5%) (Figure 12).

**Figure 13:**
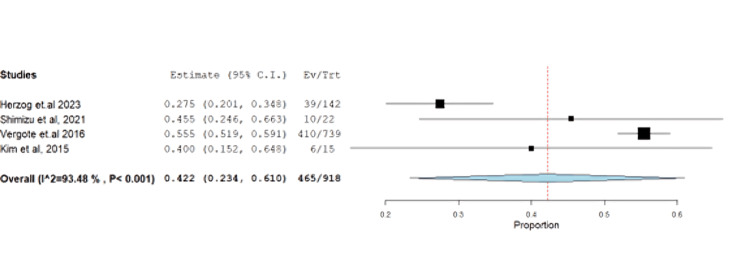
Forest plot of neutropenia.

Neutropenia had an incidence of 50.65% (95% CI: 0.23-0.61) with unresolved heterogeneity (p<0.001, I^2^=93.4%) (Figure 13).

**Figure 14:**
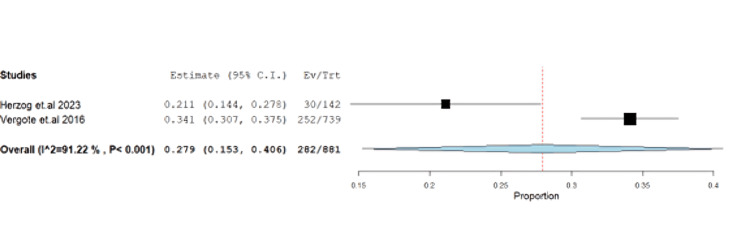
Forest plot of thrombocytopenia.

Thrombocytopenia occurred at 32% (95% CI: 0.15-0.4) with unresolved heterogeneity (p<0.001, I^2^=91.2%) (Figure 14).

**Figure 15:**
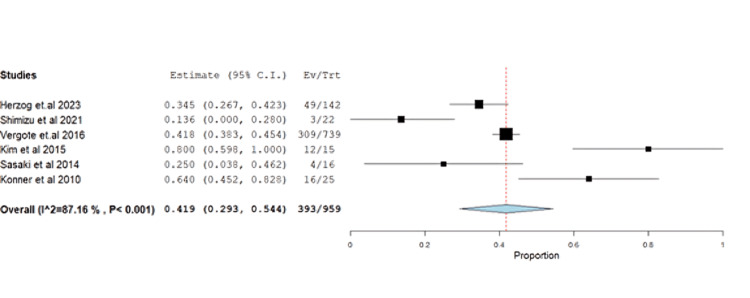
Forest plot of fatigue.

Other AEs, such as fatigue (40.98%, 95% CI: 0.29-0.54), exhibited unresolved heterogeneity (p<0.001, I^2^=87.1%) (Figure 15).

**Figure 16:**
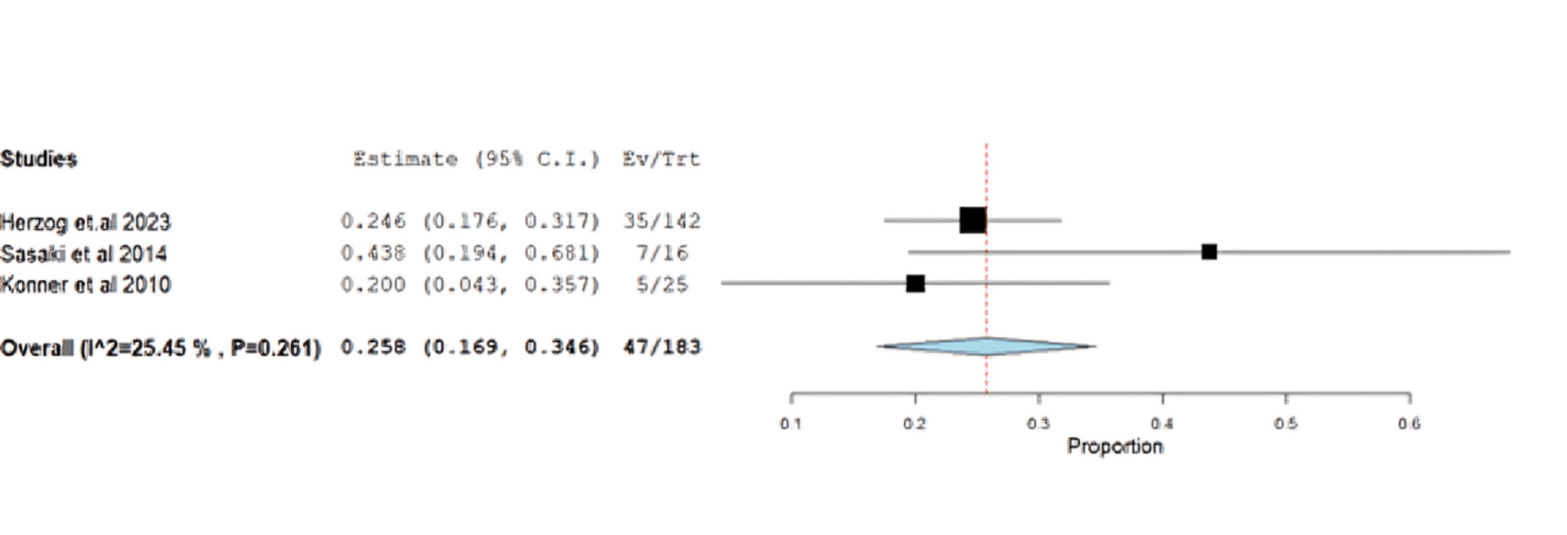
Forest plot of headache.

Headache had an incidence of 25.68% (95% CI: 0.16-0.34) with homogeneous results (p=0.26, I^2^=25.4%) (Figure 16).

**Figure 17:**
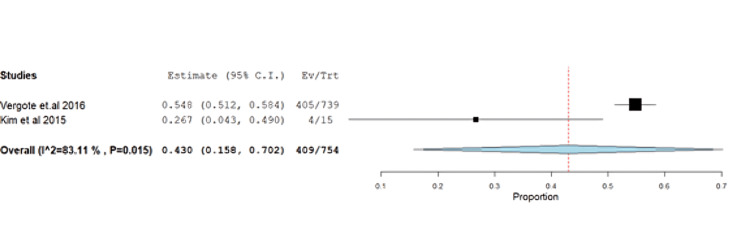
Forest plot of alopecia.

Alopecia was reported at 54.24% (95% CI: 0.09-0.23) with unresolved heterogeneity (p=0.015, I^2^=83.1%) (Figure 17).

**Figure 18:**
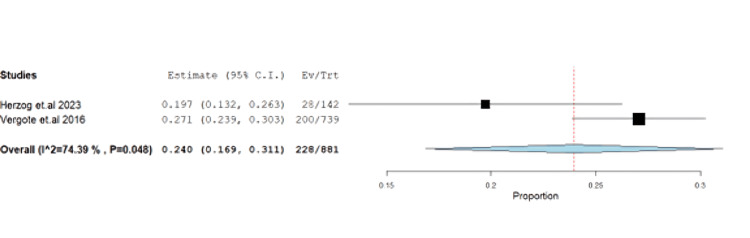
Forest plot of abdominal pain.

Abdominal pain had an incidence of 25.87% (95% CI: 0.16-0.31) with unresolved heterogeneity (p=0.048, I^2^=74.3%) (Figure 18, Table 5, and Table 6).

**Table 5:** Number of patients with AEs caused by farletuzumab. AEs: adverse effects; NA: not available

Study ID	Total	Nausea	Vomiting	Decreased appetite	Diarrhea	Constipation	Fatigue	Headache	Alopecia	Anemia	Neutropenia	Thrombocytopenia	Abdominal pain
Herzog et al., 2023 [26]	142	68	30	20	33	41	49	35	NA	54	39	30	28
Shimizu et al., 2021 [25]	736	5	2	NA	3	2	3	NA	NA	6	10	NA	NA
Vergote et al., 2016 [18]	54	404	245	170	267	228	309	NA	405	303	410	252	200
Kim et al., 2016 [22]	15	5	3	3	3	4	12	NA	4	2	6	NA	NA
Sasaki et al., 2015 [24]	25	5	NA	5	NA	NA	4	7	NA	NA	NA	NA	NA
Armstrong et al., 2013 [17]	16	NA	NA	NA	NA	NA	NA	NA	NA	NA	NA	NA	NA
Konner et al., 2010 [23]	22	NA	NA	NA	4	NA	16	5	NA	NA	NA	NA	NA

**Table 6:** Incidence of AEs caused by farletuzumab. AEs: adverse effects; SE: standard error

Variables	No. of studies	Effect estimates	Incidence %	95% CI	SE	P-value	No. of participants	Heterogeneity	
								I^2^	P-value
Nausea	5	0.413	52.14	(0.300-0.526)	0.058	<0.001	487	79.338	<0.001
Vomiting	4	0.219	30.5	(0.107-0.331)	0.057	<0.001	280	86.561	<0.001
Decreased appetite	4	0.2	21.71	(0.132-0.269)	0.035	<0.001	198	62.799	0.045
Diarrhea	5	0.232	32.87	(0.130-0.335)	0.052	<0.001	310	83.296	<0.001
Constipation	4	0.249	29.95	(0.162-0.337)	0.045	<0.001	275	74.505	0.008
Fatigue	6	0.419	40.98	(0.293-0.544)	0.105	<0.001	393	87.16	<0.001
Headache	3	0.258	25.68	(0.169-0.346)	0.045	<0.001	47	25.45	0.261
Alopecia	2	0.43	54.24	(0.092-0.237)	0.139	0.002	409	83.11	0.015
Anemia	4	0.333	39.76	(0.238-0.427)	0.048	<0.001	365	73.538	0.01
Neutropenia	4	0.422	50.65	(0.234-0.610)	0.096	<0.001	465	93.475	<0.001
Thrombocytopenia	2	0.279	32.00	(0.153-0.406)	0.065	<0.001	282	91.221	<0.001
Abdominal pain	2	0.24	25.87	(0.169-0.311)	0.036	<0.001	228	74.387	0.048

Discussion

As far as we know, this is the first comprehensive meta-analysis that provides valuable insights into the efficacy and safety of farletuzumab in ovarian cancer treatment, enhancing our understanding of its role in ovarian cancer management and guiding its integration into clinical practice more effectively.

Due to the lack of effective screening programs, ovarian cancer is diagnosed at an early stage only in about 25% of the cases, making the majority of the patients diagnosed in advanced stages. In such instances, the standard care is extensive surgical cytoreduction followed by platinum-taxane-based chemotherapy [27]. Within this standard care, a five-year survival rate for women diagnosed with ovarian cancer at stage III or IV ranges from 10% to 30% [27]. Vergote et al. conducted a noteworthy randomized controlled trial focusing on the treatment of advanced ovarian cancer. This phase III study compared two treatment approaches: primary debulking surgery followed by chemotherapy versus neoadjuvant chemotherapy followed by interval debulking surgery. The findings revealed that the median overall survival for patients in both groups was similar, with 29 months in the primary surgery group and 30 months in the neoadjuvant chemotherapy group, and this disparity was not statistically significant [28].

Roughly 70-80% of ovarian cancer patients experience relapse following first-line chemotherapy [29], highlighting a significant gap in addressing recurrent ovarian cancer, which represents an unresolved medical challenge. There is a trend toward a customized treatment strategy, as evidenced by multiple ongoing phase III trials exploring targeted therapies in combination with chemotherapy such as farletuzumab [29].

Platinum-sensitive and platinum-resistant statuses are critical considerations in managing recurrent ovarian cancer, marking divergent responses to platinum-based chemotherapy, particularly when integrated with targeted therapies like bevacizumab and farletuzumab. In a randomized phase III study involving patients with platinum-sensitive recurrent ovarian cancer, the results revealed a PFS of 11.8 months for those receiving bevacizumab compared to 8.8 months for those without [29]. Platinum-resistant ovarian cancer has a poor prognosis with limited treatment options. A phase II trial (JGOG3023) assessed adding bevacizumab to chemotherapy. The combination significantly improved PFS to four months vs. 3.1 months with chemotherapy alone (p=0.0082) [30].

Our pooled analysis of farletuzumab showed promising efficacy, with a comparable PFS to bevacizumab (10.5 months), indicating its potential impact on disease progression, especially platinum-sensitive recurrent ovarian cancer. This finding is consistent with individual studies showing improvements in PFS when farletuzumab is added to standard chemotherapy regimens [27,18]. Furthermore, farletuzumab shows promise in conferring a survival advantage, with an OS of 36.7 months, surpassing the median OS of 29-30 months observed in standard care (surgery plus chemotherapy) in the Vergote et al. trial [28]. This suggests a potential for farletuzumab to significantly improve survival outcomes in this patient population compared to conventional treatments.

On the other hand, the phase III trial evaluating farletuzumab in platinum-sensitive recurrent ovarian cancer did not demonstrate a significant improvement in PFS compared to placebo [18]. Still, subgroup analyses and previous phase II studies have suggested potential benefits in specific patient populations, such as those with lower CA-125 levels at baseline [18]. The pooled analysis corroborates these findings by demonstrating a favorable impact of farletuzumab on disease outcomes, particularly in patients with lower CA-125 levels.

The impact of farletuzumab on different disease outcomes further supports its efficacy. The high incidence of partial response (55.25%) suggests that farletuzumab has the potential to induce tumor shrinkage and promote tumor regression, which is a desirable outcome in the treatment of ovarian cancer. Additionally, the incidence of stable disease (28.68%) indicates that farletuzumab may help stabilize the disease in many patients, preventing further tumor growth and spread. These findings are consistent with preclinical data demonstrating the ability of farletuzumab to inhibit ovarian cancer cell growth [16,31].

A phase II trial evaluating the use of farletuzumab in combination with carboplatin and taxane among patients with platinum-sensitive ovarian cancer demonstrated enhanced response rates, with a 7% complete response rate and a 63% partial response rate. Furthermore, this combination therapy was associated with a prolonged time to progression, with 21% of patients experiencing a second remission longer than their initial one [28,32]. This is particularly noteworthy given the challenges associated with treating recurrent ovarian cancer, where durable responses to repeated therapy are less likely to occur [33].

The safety profile of farletuzumab is a crucial consideration in evaluating its overall benefit-risk profile [34-36,25]. The pooled analysis reveals that gastrointestinal AEs, including nausea, vomiting, diarrhea, and constipation, are prominent among patients receiving farletuzumab. While these AEs are common in cancer patients undergoing chemotherapy, their high incidence warrants close monitoring and appropriate management to ensure patient comfort and adherence to treatment [18,37].

Hematological AEs such as anemia, neutropenia, and thrombocytopenia are frequently observed with farletuzumab treatment. These findings are consistent with the known myelosuppressive effects of chemotherapy, which may necessitate dose modifications or supportive care interventions to mitigate the risk of complications such as infections and bleeding [38-40].

Other AEs reported in the pooled analysis, such as fatigue, headache, alopecia, and abdominal pain, are also consistent with the known side effect profile of chemotherapy and targeted therapies commonly used in the treatment of ovarian cancer. While these AEs may impact patients' quality of life, their management is generally well-established, and supportive care measures can help alleviate symptoms and improve treatment tolerability [17,23].

On the safety front, while gastrointestinal and hematological AEs are common, they are generally manageable and expected in the context of chemotherapy. The observed heterogeneity in AE reporting underscores the need for standardized reporting practices and further investigation into factors contributing to variability in toxicity profiles across studies.

The heterogeneity observed in Vergote et al. [18], which is the largest study to date involving 739 patients and utilizing two doses of farletuzumab (1.25 mg/kg and 2.5 mg/kg), underscores the complexity of patient responses to treatment and the need for further exploration. Given the absence of dose-limiting toxicities, the study provides a strong rationale for investigating higher dosing regimens of farletuzumab to potentially enhance its exposure-benefit effect and improve treatment outcomes for ovarian cancer patients.

Strengths and Limitations

Our paper contributes to ovarian cancer treatment, comprehensively evaluating farletuzumab's efficacy and safety. As the first systematic review and meta-analysis focused on farletuzumab, our work fills a crucial gap in the literature, consolidating existing evidence and providing valuable insights for clinical practice. By including randomized controlled studies in our analysis, we ensure a high level of methodological rigor, enhancing the reliability and validity of our findings. Additionally, our use of the ROBINS-I framework for quality assessment further strengthens the robustness of our conclusions, allowing for a nuanced evaluation of study biases and ensuring the accuracy of our synthesis. However, several limitations should be acknowledged. Firstly, the study is constrained by the limited number of included studies, which reflects the inability to access publication bias. Additionally, the lack of blinding in some of the included studies introduces potential bias and may influence outcome assessment. Moreover, unresolved heterogeneity in the outcomes across studies may impact the reliability of the pooled results. While efforts were made to address heterogeneity through subgroup analyses and sensitivity analyses, the sources of heterogeneity could not be completely elucidated. Finally, we included single-arm studies in our analysis. Consequently, findings from this study should be interpreted cautiously and may require validation through randomized controlled trials to confirm their reliability and generalizability.

## Conclusions

The study demonstrates that farletuzumab is promising in treating ovarian cancer, as evidenced by a PFS of 10.5 months and OS of 36.7 months among patients, particularly in platinum-sensitive recurrent ovarian cancer. However, further investigation, including randomized controlled trials and standardized reporting practices, is warranted to validate these findings and elucidate the optimal dosing regimen and patient selection criteria for farletuzumab therapy. The safety profile of farletuzumab appears acceptable, with AEs consistent with those commonly associated with chemotherapy. However, continued monitoring and appropriate management of AEs are essential to ensure patient comfort and treatment adherence.
